# Does practicing a wide range of joint angle configurations lead to higher flexibility in a manual obstacle-avoidance target-pointing task?

**DOI:** 10.1371/journal.pone.0181041

**Published:** 2017-07-10

**Authors:** Inge Tuitert, Reinoud J. Bootsma, Marina M. Schoemaker, Egbert Otten, Leonora J. Mouton, Raoul M. Bongers

**Affiliations:** 1 Aix-Marseille Univ, CNRS, Institut des Sciences du Mouvement, Marseille, France; 2 University of Groningen, University Medical Center Groningen, Center for Human Movement Sciences, Groningen, the Netherlands; VU University Amsterdam, NETHERLANDS

## Abstract

Flexibility in motor actions can be defined as variability in the use of degrees of freedom (e.g., joint angles in the arm) over repetitions while keeping performance (e.g., fingertip position) stabilized. We examined whether flexibility can be increased through enlarging the joint angle range during practice in a manual obstacle-avoidance target-pointing task. To establish differences in flexibility we partitioned the variability in joint angles over repetitions in variability within (GEV) and variability outside the solution space (NGEV). More GEV than NGEV reflects flexibility; when the ratio of the GEV and NGEV is higher, flexibility is higher. The pretest and posttest consisted of 30 repetitions of manual pointing to a target while moving over a 10 cm high obstacle. To enlarge the joint angle range during practice participants performed 600 target-pointing movements while moving over obstacles of different heights (5–9 cm, 11–15 cm). The results indicated that practicing movements over obstacles of different heights led participants to use enlarged range of joint angles compared to the range of joint angles used in movements over the 10 cm obstacle in the pretest. However, for each individual obstacle neither joint angle variance nor flexibility were higher during practice. We also did not find more flexibility after practice. In the posttest, joint angle variance was in fact smaller than before practice, primarily in GEV. The potential influences of learning effects and the task used that could underlie the results obtained are discussed. We conclude that with this specific type of practice in this specific task, enlarging the range of joint angles does not lead to more flexibility.

## Introduction

Skilled behavior is characterized by flexibility [1]. When attempting to avoid spilling coffee from a hand-held cup, such flexibility is for instance seen in the adaptations of the joint angles of the arm following a slight perturbation. Conceptually, flexibility may be defined as deploying a range of different solutions to solve a given motor problem. This can be operationalized to the observation of variability in the use of elemental degrees of freedom (such as joint angles) over repetitions of trials while task performance (such as holding the cup without spilling coffee) is maintained [2,3].

Because the availability of multiple solutions to a given motor problem is thus a prerequisite for flexibility, the latter capitalizes on the redundancy of elemental degrees of freedom observed at many levels of the movement-production system [1,4]. While from a computational point of view motor redundancy is generally considered a problem, requiring additional constraints to harness the system [5,6], the principle of motor abundance suggests that it is in fact a bliss [4,7,8]. Indeed, the organization of elemental variables (i.e., individual degrees of freedom) into functional units (i.e., synergies) allows variations in solutions, not only when a task is performed repeatedly within the same context [9], but also when the context changes. An increase in variability in the use of degrees of freedom not affecting performance (i.e., flexibility) has for instance been observed when participants perform a target-pointing task in the context of potential changes in target location, or in the presence of a secondary task or new constraints, or when stabilizing movements to multiple task goals simultaneously [10–17].

Practice under specific conditions has also been reported to lead to the deployment of a larger range of solutions [18–21]. Particularly interesting for the present purposes was the finding that practicing a target-pointing task in the presence of an unfamiliar force-field resulted in more flexible target-pointing behavior in a test condition without such an additional force-field [22]. If, as suggested by these authors, the observed increase in flexibility in the test condition resulted from practice under a condition requiring participants to explore a wider range of joint configurations, we hypothesized that flexibility could be increased in a manual obstacle-avoidance target-pointing task in which the hand had to move over an obstacle to reach its target location. Practicing the task with obstacles of varying height was expected to lead to variable movement trajectories of the fingertip during the reach [12,23,24]. This was hypothesized to stimulate participants to use an enlarged range of joint angles (i.e. degrees of freedom) during pointing to the (unchanged) target. The idea was thus that practicing such an enlarged range of joint angles would give rise to an increase in flexibility for pointing movements over an obstacle of middle-range height which was not included in the practice phase.

Flexibility was quantified by the uncontrolled manifold (UCM) method, a method often used in studies that start from the principle of abundance [4], which parses the total variability observed in individual degrees of freedom over repetitive trials [3,25]. When this method is applied to manual pointing movements the degrees of freedom are the joint angles of the upper limb [12,26–28]. All possible arm postures reached by moving the shoulder, elbow, wrist and finger can be captured in a multi-dimensional space where each axis corresponds to a different joint angle; this space is the joint space. Within this joint space lies a solution space for the pointing task; this solution space consists of all different joint angle configurations where the fingertip reaches the target location. Goal equivalent variability (GEV) in joint angles is defined by variability of joint angle configurations within the solution space whereas non-goal equivalent variability (NGEV) is defined by variability outside the solution space. The former type of variability does not affect the position of the fingertip, while the latter does [2,3,25]. When the variability within the solution space is larger than the variability outside the solution space, task performance over repetitions reflects flexibility. A larger GEV with respect to NGEV reflects a larger flexibility.

Thus, the rationale of the present study was the following. If practicing with obstacles of different height gives rise to the use of an enlarged range of joint angle configurations during practice, this may be expected to result in the continued deployment of multiple solutions after practice. Because the to-be-reached target position is kept similar, flexibility would thus be increased. Operationally, this corresponds to a larger increase in GEV than in NGEV after practice.

## Methods

### Participants

Thirteen right-handed participants (6 males and 7 females, mean age 23.6 years, SD 1.4 years) participated in the experimental group and nine right-handed participants (3 males and 6 females, mean age 22.8 years, SD 2.3 years) participated in the control group. All participants had normal or corrected to-normal vision.

### Ethics statement

The study was approved by the local Ethical Board of the Center for Human Movements Sciences (University Medical Center Groningen). Participants gave written informed consent before the start of the experiment.

### Materials and procedure

**Fig 1:**
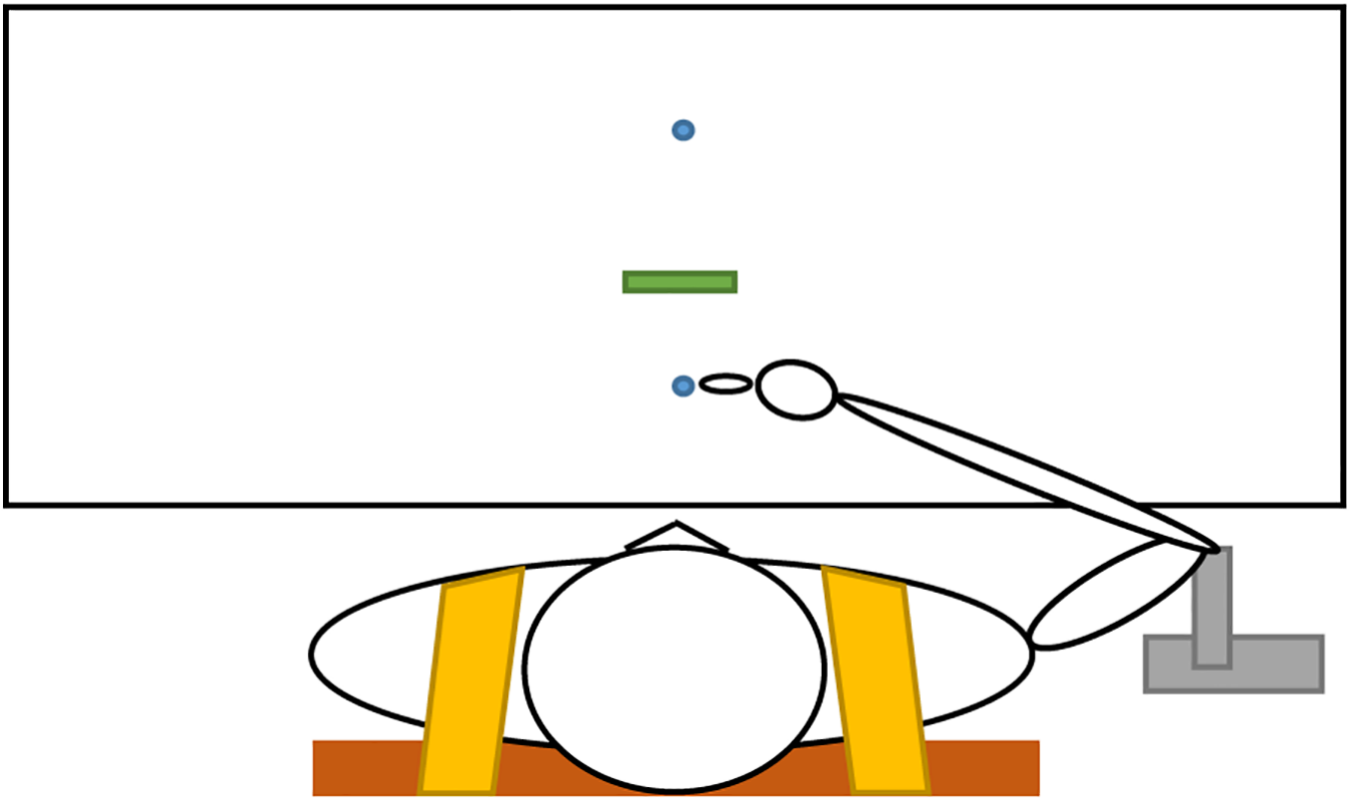
Bird’s-eye view of the set-up of the experiment where the participant is in starting position. The elbow of the participant is on the grey stand and the fingertip of the participant is on the start target. The second blue dot is the end target and the green rectangle is the obstacle. The participant is strapped to the brown chair with the yellow bandage.

Participants sat on a chair placed at a table. The backrest of the chair was extended with a plate to which the trunk of the participant was gently strapped to prevent movements of the trunk and kept the shoulder at approximately the same position in space. At the beginning of each trial, participants placed the fingertip of the right hand on the start location (i.e., a 1 cm diameter dot on the table) and held the elbow against a stand to standardize starting posture as much as possible across trials (see Fig 1). Following a ‘go’ signal delivered by the experimenter, participants performed a forward movement in the sagittal plane to reach the target (i.e., a second 1 cm diameter dot on the table) located at a 30 cm distance in front of the start position. During the movement they had to lift the index finger over an obstacle, placed at 10 cm in front of the starting position (see Fig 1). Participants were instructed to perform the movement as fast and accurate as possible but could initiate the movement at their convenience after the ‘go’ signal.

The obstacles were 11 cuboid wooden stands (3 x 1 cm ground surface dimensions) varying in height from 5 to 15 cm in steps of 1 cm. Kinematic data were collected at a sampling frequency of 100 Hz with two Optotrak 3020 system sensors (Northern Digital, Waterloo, Canada). Six rigid plates, made of PVC, with each three markers were attached to the sternum, to the acromion, at the left side of the right upper arm below the insertion of the deltoid, proximal to the ulnar and radial styloids, to the dorsal surface of the hand [29], and to the index finger [27] with skin friendly tape. Following the procedure described by Van Andel et al. [29], for each participant the positions of the six rigid bodies were linked to the 19 local anatomical positions using a standard pointer device. A small aluminum plate was taped under the index finger to prevent flexion-extension in the interphalangeal joints while allowing for flexion-extension and adduction-abduction in the metacarpal phalangeal joint [27,30].

### Design

The experiment took place over 3 consecutive days, with participants performing a pretest followed by 200 practice trials on day 1, 200 practice trials on day 2, and 200 practice trials followed by a posttest on day 3. The pre- and posttests consisted of 30 movements over a 10 cm high obstacle. Practice consisted of 600 trials over obstacles of various other heights (5, 6, 7, 8, 9, 11, 12, 13, 14, 15 cm), presented in blocks of 30 trials. With the order of presentation of the different obstacles during practice being quasi-random, participants performed 60 trials with each of the 10 practice obstacles. There was one minute of rest between the tests and practice blocks. A no-practice control group only performed the pretest on day 1 and the posttest on day 3.

### Data analysis

The data were analyzed using customized programs written in Matlab (Mathworks, Natick, MA). Converting the collected kinematic data for the 6 rigid bodies (marker clusters) to the positions of 19 anatomical landmarks [29] allowed extraction of the time series of the fingertip position and 9 relevant joint angles (see below).

### End-effector

X-Y-Z velocities were derived using the three-point central difference method. Tangential velocity was calculated at each point in time as the square root of the sum of the three squared velocities. For each trial, the start and the end of the movement were determined by searching backward and forward, respectively, from the moment at which peak tangential velocity was reached. The start of the movement was identified as the first data point (working backwards from the moment of peak velocity) where the tangential velocity fell below a threshold of 5 cm/s, and the position in the forward direction and the position in the sideward direction was within the boundary of 1.5 cm from the center of the start point. The end of the movement was identified as the first data point where the vertical position of the fingertip was at table level. Movement time was determined by the time period between the start and the end of the movement. Peak velocity was defined as the maximum value in the tangential velocity profile. The symmetry index of velocity was defined as the time from movement onset to moment of peak velocity divided by total movement time where 0.5 denotes a symmetric velocity profile. The moment the fingertip crossed the obstacle was identified as the first data point where the position of the fingertip in the forward direction passed the obstacle location at 10 cm in front of the start position. End-point precision was calculated as the within-participant SD per condition (pretest, practice, posttest) of the position of the fingertip in the forward and sideward directions at the end of the movement.

### Joint angles

Joint rotations were calculated following the orientations as proposed in the ISB standardization proposal for the upper extremity by Wu et al. [31]: shoulder plane of elevation (SPE), shoulder elevation (SE), shoulder inward–outward rotation (SIO), elbow flexion–extension (EFE), forearm pronation–supination (FPS), wrist flexion–extension (WFE), wrist abduction–adduction (WAA), index finger flexion–extension (FFE), and index finger abduction–adduction (FAA). We determined the arm’s postural configuration on each individual trial (i) at the moment the fingertip passed over the obstacle and (ii) at the end of the reaching movement. We specifically chose to analyze the postural configurations at these instants because moving over the obstacle and reaching the target were the two prominent constraints of the task. From the variations in postural configurations observed at these instants over repeated trials during the pretest, practice, and posttest we derived the within-participant ranges and variances for each of the nine joints. The measure of joint angle range was operationally defined as the within-participant mean over the observed ranges of the nine joints (collapsed over obstacles in the practice phase). The measures of joint angle variance were calculated per obstacle, and averaged over obstacles and joints when analyzing the practice phase (not collapsed over obstacles).

### Uncontrolled manifold analysis

The uncontrolled manifold (UCM) method based on the covariance matrix (C) [32] was used to determine flexibility when the fingertip was at the obstacle and at the end of the movement. The elemental variables selected in this task were the joint angles of the shoulder, elbow, wrist, and finger (9-DOF). The performance variable selected was the fingertip position of the index finger (3-DOF). The relations between changes in joint angles (i.e., postural configurations) and fingertip positions were computed and united in a Jacobian matrix (J) [26,32]. The null-space of the J was used as a linear approximation of the UCM. The variance components GEV and NGEV were computed by projecting the total variance in joint space onto the null-space of J and the orthogonal complement, respectively. Each UCM (GEV and NGEV) component was normalized by its number of DOFs.

Eqs 1 and 2 show the computation of GEV and NGEV, where tr denotes the trace of a matrix, null denotes the null space of a matrix, t denotes the transpose of a matrix, denotes the orthogonal of a matrix, C denotes the covariance matrix of all joint angles, n denotes the dimension of the joint space (n = 9), and d denotes the dimension of the task space (d = 3). The end-effector was considered flexibly stabilized when RATIO was larger than 0.5 (Eq 3). To correct for non-normal data distributions GEV, NGEV and RATIO were log transformed prior to the statistical analysis [32], indicated by the subscript log. In order to allow comparison with other studies, figures in the results section display non-transformed data.

$$GEV=\frac{{tr(null(J)}^{t}*C*null(J))}{n-d}$$ Eq (1)

$$NGEV=\frac{{tr(((J^{t})}^{⊥}{)}^{t}*C*(J^{t}{)}^{⊥})}{d}$$ Eq (2)

$$RATIO=\frac{GEV}{GEV+NGEV}$$ Eq (3)

### Statistical analysis

SPSS software (IBM, Armonk, New York) was used to perform the statistical analyses. Five types of analysis were performed. First, to examine the influence of obstacle height on the maximum height of the fingertip during the pointing movement we performed a linear regression with obstacle height as dependent variable and maximum height of the fingertip as independent variable on the trials of the practice phase. Second, to examine whether practice with multiple obstacles gave rise to an increase in the range of joint angles, we compared the range of joint angles in the pretest and during practice of the experimental group. A repeated-measures ANOVA on the range of joint angles was performed with Condition (pretest and practice) and Instant (at obstacle and end of movement) as within-participant factors. Third, to examine whether practicing moving over different obstacles affected moving over any individual obstacle during the practice phase we first determined joint angle variance, GEV, NGEV and RATIO for each individual obstacle. After averaging over obstacles, joint angle variance, GEV_log_, NGEV_log_, and RATIO_log_ were submitted to repeated-measures ANOVAs with Condition (pretest and practice), and Instant (at obstacle and end of movement) as within-participant factors. Fourth, to examine whether joint angle variability increased from the pretest to the posttest the joint angle variances observed during the pretest and the posttest of the experimental and control group were compared. A repeated-measures ANOVA on joint angle variance was performed with Group (experimental group and control group) as between-participant factor and Condition (pretest and posttest) and Instant (at obstacle and end of movement) as within-participant factors. Finally, to establish how joint angle variability affected end-effector position and how practice affected flexibility, repeated-measures ANOVAs were performed on GEV_log_, NGEV_log_, and RATIO_log_ with Group (experimental group and control group) as between-participant factor and Condition (pretest and posttest), and Instant (at obstacle and end of movement) as within-participant factors. In all ANOVAs Greenhouse-Geisser corrections were applied when the assumption of sphericity was violated. The level of significance was set to α = 0.05. Where appropriate, significant main effects and interactions were further analyzed using Newman-Keuls post-hoc tests. Effect sizes were calculated using generalized eta-squared statistics [33,34] and interpreted according to Cohen’s recommendation of 0.02 for a small intensity effect, 0.13 for a medium intensity effect, and 0.26 for a large intensity effect [35]. Significant results with effect sizes smaller than 0.02 are not discussed.

## Results

### Trial removal

Of the total of 9120 trials recorded, 42 (i.e., 0.5%) were discarded due to (partial) occlusion of markers.

### End-effector kinematics

End-effector kinematics were calculated to examine whether these measures were in line with other studies with a similar task. In line with [24], maximum height of the fingertip (MHFtip) co-varied with obstacle height (OH) as confirmed by a linear regression analysis (*F*(1, 128) = 1868, *p* < 0.001, with, *r*^*2*^ = 0.94). For both groups the variability of fingertip position at the end of the movement, the measure used for end-point precision, was systematically small on both forward and sideward directions compared to the 1 cm target diameter (Table 1). This result indicated that the participants performed precise movements. Table 1 also shows the means and standard errors of the means of movement time, symmetry index of velocity, and peak velocity for the pretest, practice and posttest of the experimental group. We qualitatively compared these outcomes to another experiment from our lab where similar movements were made without an obstacle (unconstrained condition of [36]). The comparison indicated that movements over an obstacle have a higher movement time and that peak velocity occurred earlier in movement over an obstacle compared to movements without obstacle which is in agreement with earlier studies [24]. However, contrary to the findings of the latter study, here the peak velocity was lower compared to the study without obstacles [36].

**Table 1:** End-effector kinematics per condition and group.

Dependent variable	Group	Pretest	Practice	Posttest
SD forward direction (cm)	Experimental	0.32 (0.036)	0.32 (0.018)	0.30 (0.02)
	Control	0.43 (0.05)	-	0.43 (0.02)
SD sideward direction (cm)	Experimental	0.28 (0.02)	0.30 (0.018)	0.28 (0.02)
	Control	0.35 (0.032)	-	0.37 (0.01)
Movement time (s)	Experimental	0.73 (0.04)	0.65 (0.03)	0.69 (0.02)
	Control	0.55 (0.04)	-	0.52 (0.04)
Symmetry index of velocity	Experimental	0.35 (0.03)	0.42 (0.03)	0.37 (0.03)
	Control	0.43 (0.03)	-	0.45 (0.03)
Peak velocity (cm/s)	Experimental	106.9 (5.0)	112.3 (5.1)	99.7 (4.0)
	Control	134.5 (8.5)	-	138 (7.9)

Means and standard errors of the means (between parentheses) of the SD of fingertip position at the end of the movement in the forward direction and the sideward direction, movement time, symmetry index of velocity and peak velocity for each condition and group.

We also observed a different speed-accuracy trade-off (see also [37,38]) between the experimental and the control group, as the control group was less accurate and faster compared to the experimental group. These findings indicate slight differences in strategy between our experimental group and the control group.

### Range of joint angles in the pretest and during practice (collapsed over obstacles)

**Fig 2:**
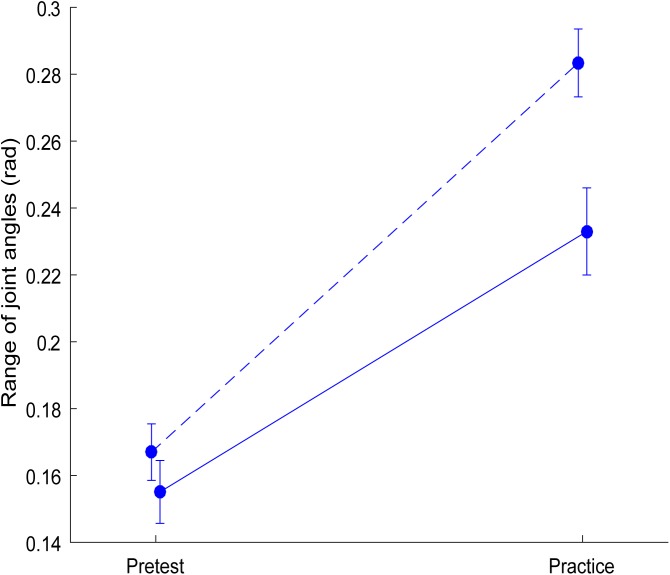
Range of joint angles for each Instant and Condition for the experimental group. The dotted blue line corresponds with Instant at the obstacle and the solid blue line corresponds with Instant at the end of the movement. The pretest is presented on the left and practice on the right. The error bars display the standard error of the mean.

Analysis of joint angle range in the pretest and during practice of the experimental group revealed significant main effects of Condition (*F*(1,12) = 95.23, *p* < 0.001, *η*^2^_g_ = 0.81) and Instant (*F*(1,12) = 15.37, *p* = 0.002, *η*^2^_g_ = 0.31), as well as a significant interaction between the two (*F*(1,12) = 13.34, *p* = 0.003, *η*^2^_g_ = 0.15). Post-hoc analysis of the interaction demonstrated that practice with obstacles of different heights gave rise to an increase in joint angle range, both at the obstacle and at the end of the movement (see Fig 2). During the pretest joint angle range did not significantly differ (*p* = 0.14) between the two instants, while during the practice session the range was significantly larger at the obstacle than at the end of the movement (*p* < 0.001). Overall, we conclude that the practice session with obstacles of different height seems to do what we intended it to do: it evoked an enlarged range of joint angles at both instants.

### Joint angle variance and flexibility in the pretest and during practice (not collapsed over obstacles)

**Fig 3:**
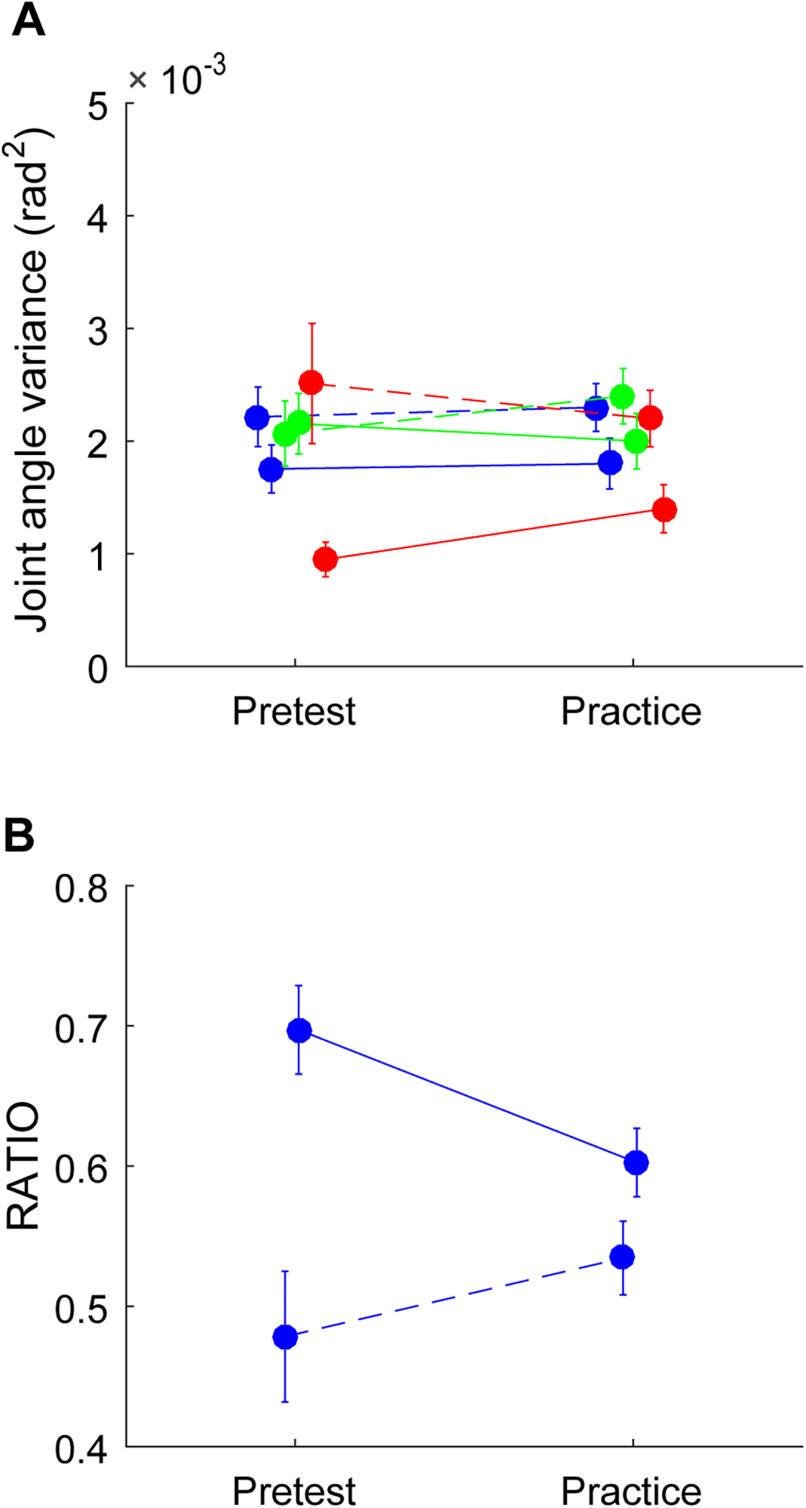
Joint angle variance, GEV, NGEV and flexibility for each Instant in the pretest and during practice per obstacle. Panel A depicts joint angle variance in blue, GEV in green and NGEV in red and panel B depicts RATIO. In each panel the dotted lines correspond with Instant at the obstacle while the solid lines correspond with Instant at the end of the movement. The pretest is presented on the left and practice on the right. The error bars display the standard error of the mean.

We recall that for this specific analysis the dependent variables concerning the practice phase were first calculated for each individual obstacle and then averaged over obstacles, instead of being collapsed over obstacles, as in the previous sections. The results of the repeated-measures ANOVAs with the within-participant factors Condition (pretest and practice) and Instant (at the obstacle and at the end of movement) are summarized in Table 2. The analysis of joint angle variance only revealed a medium-intensity main effect of Instant. While GEV_log_ was not systematically affected, the effect of joint angle variance was mirrored in a strong-intensity effect of Instant for NGEV_log_. These effects indicated that the joint angle variance, and specifically NGEV_log_, was larger at the obstacle than at the end of movement (see Fig 3). The analysis on RATIO_log_ revealed not only a main effect of Instant but also an interaction between Instant and Condition (see Table 2). Post-hoc analysis of the interaction revealed that in the pretest RATIO_log_ was lower at the obstacle compared to at the end of the movement (*p <* 0.001); no significant difference was found during practice. Thus, contrary to our expectations, the joint angle variance, GEV_log_ and RATIO_log_ were not larger during practice compared to the pretest. Taken together, these analyses indicated that an enlarged joint angle range does not lead to a higher GEV_log_ or to a higher flexibility per obstacle during practice for any of the two instants.

**Table 2:** Summary of ANOVA results on variance of joint angles (JA), GEV_log_, NGEV_log_, and RATIO_log_ with factors Instant (at the obstacle and at the end of movement) and Condition (pretest and practice).

	Within/between subjects factor	*F*	*p*	*η*^2^_g_	DoF (factor/error)
Variance of JA	Instant	8.28	0.014	0.17	1/12
	Condition	0.12	0.74	<0.02	1/12
	Instant x Condition	0.005	0.95	<0.02	1/12
GEV_log_	Instant	0.48	0.5	0.01	1/12
	Condition	0.05	0.82	<0.02	1/12
	Instant x Condition	4.52	0.055	0.05	1/12
NGEV_log_	Instant	59.57	<0.001	0.46	1/12
	Condition	0.57	0.47	0.03	1/12
	Instant x Condition	4.32	0.06	0.09	1/12
RATIO_log_	Instant	28.95	<0.001	0.36	1/12
	Condition	0.03	0.86	<0.02	1/12
	Instant x Condition	7.89	0.02	0.14	1/12

### Joint angle variance and flexibility in the pretest and posttest

**Fig 4:**
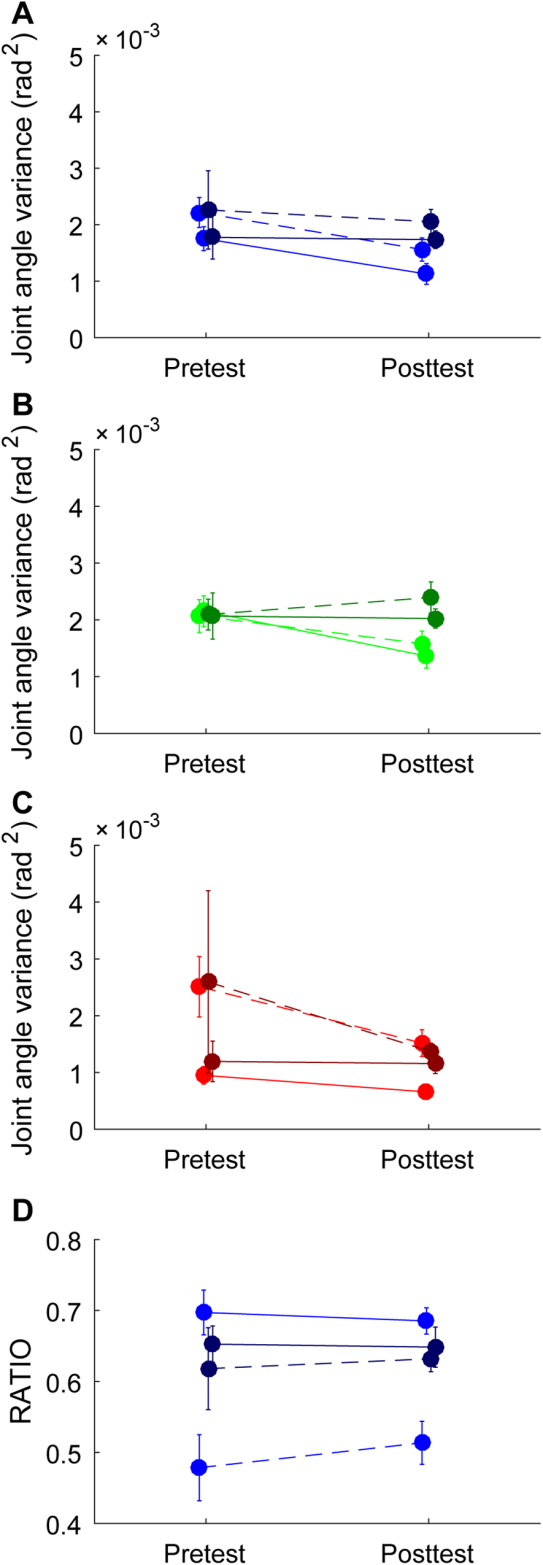
Joint angle variance, GEV, NGEV and flexibility for each Instant and Condition. Panel A depicts joint angle variance, panel B GEV, panel C NGEV, and panel D RATIO. In each panel the dotted lines correspond with Instant at the obstacle while the solid lines correspond with Instant at the end of the movement, and the light colors correspond with the experimental group while the dark colors correspond with the control group. The pretest is presented on the left and the posttest on the right. The error bars display the standard error of the mean.

To examine our hypothesis that joint angle variability would increase after practice we compared the joint angle variances of the pretest to the posttest. As can be seen from the ANOVA results reported in Table 3, comparison of the joint angle variances during the pre- and posttests only revealed a small-intensity effect of Instant (Fig 4A, compare left side of the graph with the right side), indicating that for both groups the joint angle variance was larger at the obstacle than at the end of the movement. We did not find the expected interaction effect of Condition x Group, thereby invalidating our hypothesis that the experimental group would reveal an increase in joint angle variance from pretest to posttest. We note that joint angle variance per joint did not differ much, as shown in S2.

**Table 3:** Summary of ANOVA results on variance of joint angles (JA), GEV_log_, NGEV_log_, and RATIO_log_ with factors Group (control and experimental), Instant (at the obstacle and at the end of movement), and Condition (pretest and posttest).

	Within/between subjects factor	F	p	*η*^2^_g_	DF (factor/error)
Variance of JA	Group	0.87	0.36	0.02	1/20
	Instant	9.25	<0.01	0.04	1/20
	Instant x Group	0.03	0.87	<0.02	1/20
	Condition	2.49	0.13	0.04	1/20
	Condition x Group	1.13	0.30	<0.02	1/20
	Instant x Condition	0.19	0.67	<0.02	1/20
	Instant x Condition x Group	0.09	0.76	<0.02	1/20
GEV_log_	Group	2.64	0.12	0.05	1/20
	Instant	1.80	0.19	<0.02	1/20
	Instant x Group	0.31	0.59	<0.02	1/20
	Condition	2.59	0.12	<0.02	1/20
	Condition x Group	7.82	0.011	0.05	1/20
	Instant x Condition	1.77	0.20	< 0.02	1/20
	Instant x Condition x Group	0.47	0.50	< 0.02	1/20
NGEV_log_	Group	0.12	0.73	< 0.02	1/20
	Instant	34.61	<0.001	0.23	1/20
	Instant x Group	12.71	0.002	0.10	1/20
	Condition	1.07	0.31	0.02	1/20
	Condition x Group	2.47	0.13	0.05	1/20
	Instant x Condition	0.01	0.95	< 0.02	1/20
	Instant x Condition x Group	0.02	0.90	< 0.02	1/20
RATIO_log_	Group	2.24	0.15	0.03	1/20
	Instant	28.02	<0.001	0.19	1/20
	Instant x Group	13.88	0.001	0.11	1/20
	Condition	0.50	0.49	< 0.02	1/20
	Condition x Group	0.02	0.88	< 0.02	1/20
	Instant x Condition	1.56	0.22	< 0.02	1/20
	Instant x Condition x Group	0.05	0.82	< 0.02	1/20

The unexpected finding that joint angle variance did not increase from pretest to posttest was mirrored in the GEV_log_ data: a small-intensity Condition x Group interaction (Table 3, Fig 4B) even indicated that for the experimental group GEV_log_ was lower on the posttest than on the pretest whereas it did not change for the control group. While not significantly affected by Condition (i.e., pre- or posttest), NGEV_log_ showed a medium-intensity main effect of Instant and a small-intensity interaction of Instant x Group (Table 3, Fig 4C). Analysis of the overarching interaction indicated that for both groups NGEV_log_ was higher at the obstacle than at the end and that NGEV_log_ at the end was lower for the experimental group than for the control group. Hence, the larger joint angle variance at the obstacle was mainly due to the larger NGEV_log_ at the obstacle. Finally, we examined flexibility as defined by RATIO_log_. Similar to the NGEV_log_ findings, RATIO_log_ showed a medium-intensity main effect of Instant and a small-intensity interaction of Instant x Group (Table 3, Fig 4D). The post-hoc analysis on the interaction of Instant x Group indicated that flexibility was higher at the obstacle than at the end and that this was mainly the case in the experimental group. Importantly, we did not find an increase in flexibility after practice.

In sum, these results indicate that the control group did not show any significant differences between the pretest and posttest. The experimental group, on the other hand, demonstrated a slight decrease in GEV_log_ from the pretest to the posttest, at the moment of crossing the obstacle and at the end of the movement. While flexibility was higher at the obstacle than at the end of the movement, neither NGEV_log_ nor flexibility evolved to any significant degree from the pretest to the posttest.

## Discussion

The aim of the current study was to examine whether flexibility increased through practicing an enlarged range of joint angles in a manual obstacle-avoidance target-pointing task. The idea was inspired by the principle of motor abundance and experimental studies showing that flexibility increased as a function of task constraints [7,8,10,18]. Flexibility was operationalized as the relation between variabilities in degrees of freedom over repetitions that did not (GEV) or did (NGEV) affect performance (i.e., end-effector position). During practice, participants performed movements over obstacles of varying heights (cf. [12]), which was expected to evoke deployment of an enlarged range of joint angles. This was hypothesized to lead to a higher variability in joint angles and, therefore, to a higher flexibility after practice. More precisely, flexibility would increase because of a larger increase in GEV than in NGEV after practice, reflecting the joint angle variability within and outside the solution space of the task in the joint space, respectively.

Our results demonstrated that the range of joint angles was indeed larger during practice than during the pretest. However, the enlarged range of joint angles did not lead to a higher joint angle variance or higher flexibility during practice at each obstacle. In line with this finding and contrary to our hypotheses, we did not find an increase in joint angle variance and flexibility after practice; we even observed a decrease in joint angle variance after practice. More specifically, GEV was significantly lower after practice while NGEV showed no significant change. The decrease in GEV was small; therefore, no significant change in flexibility (i.e., operationalized as GEV/(GEV+NGEV)) was found. These findings imply that although participants explored an enlarged range of joint angles during practice, they did not show more variance during and after practice than before practice. In the posttest participants performed the task successfully with less variability in the joint angles. We expected the strongest effect at the moment the obstacle was passed because during practice the range of joint angles was most directly affected at this instant. However, we also did not find a change in flexibility at the moment the obstacle was passed. In short, our findings did not support our hypothesis.

The decrease in GEV from pretest to posttest can be explained from the idea that learning occurred. In the current experiment participants repeatedly performed movements to the same target location. The trajectory to this target was different during practice due to the different obstacle heights imposed; however, the target location was similar. Because of this similar target location throughout the entire experiment, participants may just have learned to point to this target location. How might this explain our results? The finding of a decrease in GEV from pretest to posttest can be related to previous studies based on the principle of motor abundance focusing on learning, which also reported a decrease in GEV over learning [26,39,40], that is, during the practice phase. However, these previous studies also reported that NGEV as well as end-effector error decreased during practice [26,39,40]. The fact that in the present study we did not replicate these findings on NGEV is likely due to differences in the tasks studied. In our task participants already performed quite accurate and precise movements at the start of the experiment. A reason for only finding a decrease in GEV could thus be related to a floor effect in this familiar task where there was little room to decrease NGEV. Practice may have led to the emergence of more consistent movement patterns [26,41]. Following this line of reasoning, the decrease in GEV might indicate that learning occurred at the joint level.

An alternative, but related explanation could be that participants did not just learn to use a synergy to reach to the goal, but in fact used a different synergy for each obstacle. Because the task had two main constraints, that is, reaching over the obstacle and ending up at the target, it might have been the case that different synergies were instantiated for each obstacle. If each obstacle was passed using a different synergy, then this might explain why the enlarged range of joint angles over obstacles did not transfer to an enlarged range of joint angles employed in the post test, where a different obstacle was used.

The idea that the task was familiar could also be a reason for not finding changes in flexibility. Earlier experiments revealing an increase in flexibility included more challenging conditions, such as performing a secondary task, handling new constraints, stabilizing multiple task goals simultaneously, or responding to perturbations [12,10,11,13–17]. However, we specifically opted for this not very challenging task because if we had found an effect in this task, the effect would have been more likely to generalize to other tasks. A second motivation for choosing this particular task originated from a rehabilitation perspective. Flexibility is reduced in particular groups, such as persons with Down Syndrome [42,43], and neurological patients [44,45]. If flexibility had increased in our experimental task, implementing the practice phase of the current study in a rehabilitation setting would have been rather straightforward. Therefore, although the choice of the present obstacle-avoidance target-pointing task was not without purpose, its insufficiently-challenging character might have been a reason for not finding a change in flexibility.

In conclusion, the current study examined whether flexibility increased through practicing an enlarged joint angle range in a manual obstacle-avoidance target-pointing task. Contrary to our expectations, the results demonstrated that joint angle variability did not increase during practice and even decreased after practice, whereas flexibility was similar during practice and after practice. This implies that for this specific task with this specific type of practice, enlarging the range of joint angles does not lead to more flexibility.

## Supporting information

S1 File Kinematic and UCM measures. The third tab gives the explanation of the abbreviations.
(XLSX)

S2 File Joint angle variance per joint for each Instant and for the pretest and posttest. (DOCX)
