# Supplementary material for: Does practicing a wide range of joint angle configurations lead to higher flexibility in a manual obstacle-avoidance target-pointing task?
Source: PLoS One. 2017 Jul 10;12(7):e0181041. doi: 10.1371/journal.pone.0181041 (PMC5507288; doi:10.1371/journal.pone.0181041)
Supplement: S2 File — (DOCX) [file pone.0181041.s002.docx]

**Supplementary material 2**

The within-participant joint angle variance is presented for each joint in Fig. S1, at the moment the finger crossed the obstacle and at the moment of arrival at the target.


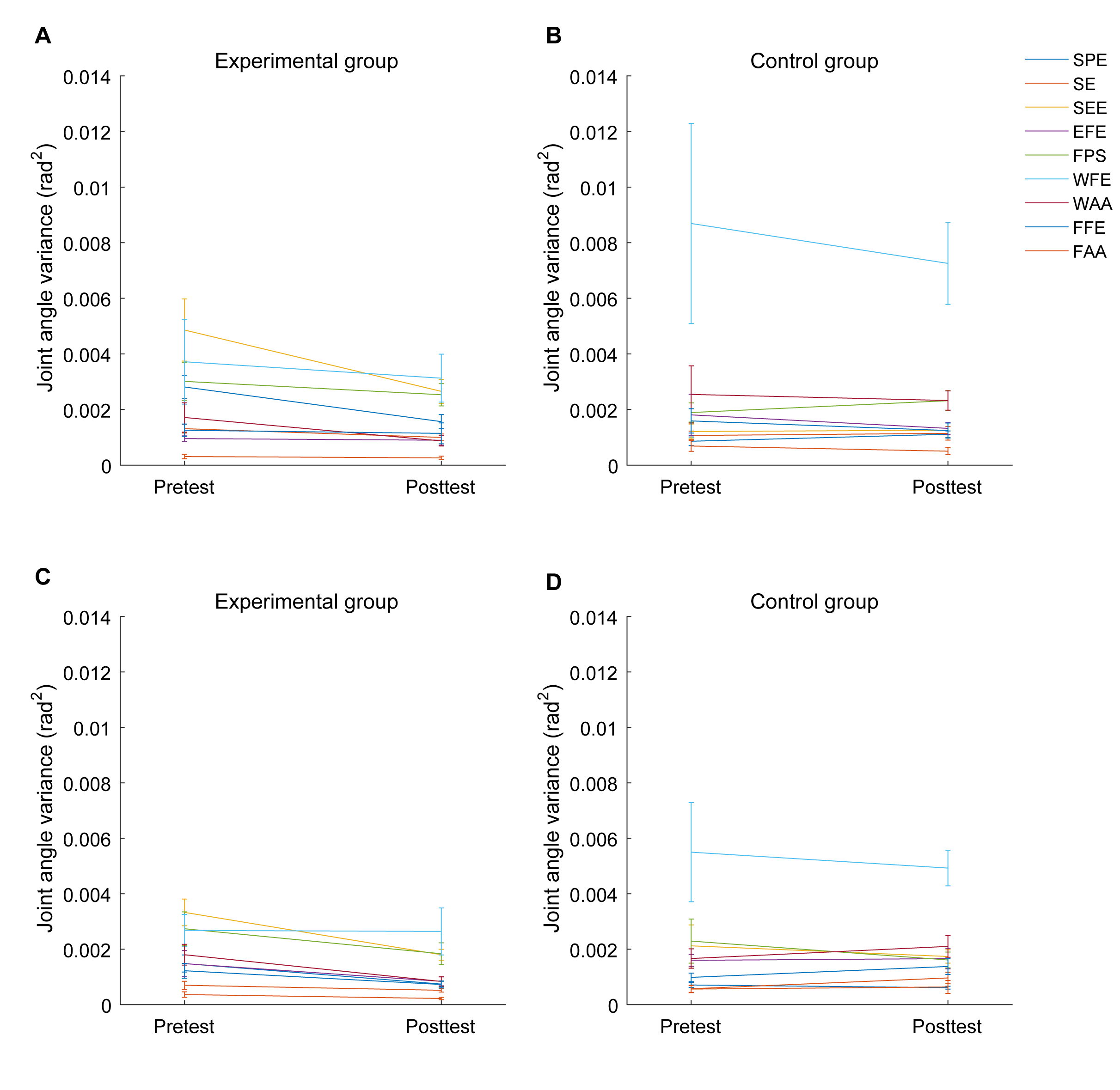


Figure S1. *Joint angle variance for each joint for the pretest and posttest (A, B) at the moment the finger crossed the obstacle and (C, D) at the moment of arrival at the target.* The abbreviation of the legend stand for: SPE: shoulder plane elevation, SE: shoulder elevation, SIO: shoulder outward inward rotation, EFE: elbow flexion extension, FPS: forearm pronation supination, WFE: wrist flexion extension, WAA: wrist abduction adduction, FFE: finger flexion extension, FAA: finger abduction adduction. The error bars indicate the standard error of the mean.
